# Current status of antisense RNA-mediated gene regulation in *Listeria monocytogenes*

**DOI:** 10.3389/fcimb.2014.00135

**Published:** 2014-09-30

**Authors:** Tilman Schultze, Benjamin Izar, Xiaoxing Qing, Gopala K. Mannala, Torsten Hain

**Affiliations:** ^1^Department of Medicine, Institute of Medical Microbiology, Justus-Liebig University GiessenGiessen, Germany; ^2^Department of Medicine, Massachusetts General Hospital and Harvard Medical SchoolBoston, MA, USA

**Keywords:** *Listeria monocytogenes*, antisense RNA, asRNA, regulation, next generation sequencing, bacteria

## Abstract

*Listeria monocytogenes* is a Gram-positive human-pathogen bacterium that served as an experimental model for investigating fundamental processes of adaptive immunity and virulence. Recent novel technologies allowed the identification of several hundred non-coding RNAs (ncRNAs) in the *Listeria* genome and provided insight into an unexpected complex transcriptional machinery. In this review, we discuss ncRNAs that are encoded on the opposite strand of the target gene and are therefore termed antisense RNAs (asRNAs). We highlight mechanistic and functional concepts of asRNAs in *L. monocytogenes* and put these in context of asRNAs in other bacteria. Understanding asRNAs will further broaden our knowledge of RNA-mediated gene regulation and may provide targets for diagnostic and antimicrobial development.

## Introduction

*Listeria monocytogenes* is a Gram-positive, facultative foodborne pathogen that causes a severe life-threatening disease (listeriosis) in susceptible humans and animals. Complex regulatory mechanisms allow *L. monocytogenes* to adapt and survive in a wide range of environmental conditions (e.g., low temperature, high pH, and high-salt conditions) and infect a variety of hosts including mammalia and insects (Cossart and Toledo-Arana, 2008). Furthermore, *L. monocytogenes* was used as a model pathogen for the investigation of key elements of cell mediated immunity (Witte et al., 2012). Given its implications as public health concern, versatility as a bacterium and experimental model, significant effort has been undertaken to characterize genomic and transcription regulation in *L. monocytogenes* (Cossart and Lebreton, 2014).

Genomic studies uncovered crucial genes regulating listerial pathogenesis, such as the ~9 kb virulence gene locus *Listeria* pathogenicity island-1 (LIPI-1) in which the major virulence determinants are organized (Chakraborty et al., 2000; Glaser et al., 2001). However, interpretation of genome-wide gene regulation in *Listeria* remains challenging due to the complex regulatory networks that are controlled by transcription regulators and alternative sigma factors (e.g., PrfA, σ^B^, and CodY) (Chaturongakul et al., 2011; Lobel et al., 2012; Xayarath and Freitag, 2012).

The recent discovery of the presence of non-coding RNA (ncRNA) elements in various bacterial genomes added a further layer of complexity in our understanding of bacterial gene regulation. In the last decade a myriad of non-coding RNAs (ncRNAs) of different genomic origin, length, function, and mechanisms of gene regulation were identified (Gottesman and Storz, 2011; Storz et al., 2011; Caldelari et al., 2013).

Although ncRNAs represent a heterogeneous group, they can roughly be divided into three categories. The first category consists of regulatory elements that are located in the 5′UTR of their targets (e.g., riboswitches, thermosensors, or pH-sensors). An important example in *L. monocytogenes* is a thermosensor that controls the major virulence regulator PrfA of LIPI-1. At low temperatures (~30°C) the thermosensor forms a complex secondary structure that prevents translation of PrfA by interfering with the Shine-Dalgarno (SD) region (Johansson et al., 2002).

*Trans*-encoded small RNA (sRNA) could be considered as the second category. Those transcripts regulate genes located elsewhere on the genome and share only limited complementarity with the target. They often interact with multiple different target transcripts, and therefore function analogous to human microRNA (Gottesman, 2005). To date, 154 sRNA were identified in the genome of *L*. *monocytogenes* and primarily termed as rli (Mandin et al., 2007; Wurtzel et al., 2012).

The last group of ncRNAs, designated as *cis*-encoded antisense RNAs (asRNAs), is located on the opposite DNA strand of their target and therefore share a high degree of complementarity with it. There is growing evidence that asRNAs are present in several Gram-positive and Gram-negative bacterial species and families with a high variability in prevalence and genomic density (Georg and Hess, 2011). The fraction of genes with a reported asRNA varies significantly with ~75% in cyanobacterium *Prochlorococcus* (Voigt et al., 2014), ~46% in *Helicobacter pylori* (Sharma et al., 2010) compared to ~20% in *Escherichia coli* (Georg and Hess, 2011).

In this review, we focus on the current status of reported asRNAs in *L. monocytogenes*, their function and outline mechanisms where applicable. A general review of the function of ncRNAs in *Listeria* is outside of the scope of this review and is summarized elsewhere (Izar et al., 2011; Cossart and Lebreton, 2014).

## Identification of asRNAs in *L. monocytogenes*

The reliable detection of antisense RNA is challenging because of technical difficulties. A major problem using microarrays, for instance, is artificially generated products during cDNA synthesis from RNA (Perocchi et al., 2007). Recently, major technical developments for generating and analyzing high-throughput data contributed to an increase in quantity and quality of information on asRNA. Until 2009, only a few asRNAs were described for *L. monocytogenes* by means of classical methods (Mandin et al., 2007). With the advent of whole genome tiling arrays and next-generation sequencing methods the number of asRNAs expanded exponentially (Toledo-Arana et al., 2009; Mraheil et al., 2011; Wurtzel et al., 2012; Behrens et al., 2014). Toledo-Arana et al. identified 21 novel asRNAs as well as 50 sRNAs (defined as <500 nucleotides), including seven that were located on the opposite strand of another transcript (Toledo-Arana et al., 2009). Applying a whole genome tiling array approach, this group investigated transcription profiles in several settings, such as growth of *L. monocytogenes* in different phases (exponential and stationary phase), distinct media, and organs (rich media, blood, and intestine) and under stress conditions (hypoxia and low temperature). This study demonstrated the influence of regulatory RNAs in response to different microenvironments.

Using 454 pyrosequencing, Mraheil et al. revealed a large portion of known regulatory RNAs. In total the 150 discovered regulatory RNA elements, of which 71 were previously unknown, include 29 asRNAs (Mraheil et al., 2011). Comparing expression profiles of extracellular bacteria to that in the intracellular compartment of murine macrophages, the authors found differential expression of asRNAs. This observation supports the notion that expression of regulatory RNAs (such as asRNAs) changes in response to extrinsic stimuli and therefore contribute to an adaptive expression program.

Another next generation sequencing platform, namely Illumina was used by Wurtzel and colleagues. In a RNA-seq experiment with transcription start site (TSS)-detection they identified 86 additional ncRNAs, including 50 novel asRNAs (Wurtzel et al., 2012). Comparing the transcriptome of *L. monocytogenes* with the closely related non-pathogenic *Listeria* species, the authors found significant divergence in the repertoire of regulatory RNAs. Furthermore, this study identified long asRNAs that are complimentary to genes but also function as sense transcripts for divergently oriented genes. Those unprecedented constructs were named “excludons” (Wurtzel et al., 2012).

The last study to date was performed by Behrens and colleagues. Using the SOLiD ultra deep sequencing platform and choosing similar conditions as Mraheil et al. (2011), 90% of known regulatory RNAs were confirmed and additional nine asRNAs were identified (Behrens et al., 2014). Moreover, four asRNAs previously described (Toledo-Arana et al., 2009; Wurtzel et al., 2012) were confirmed in this study and—likely as a consequence of higher coverage rate—were predicted to be even longer than initially reported.

In summary, using different array and sequencing methods more than hundred asRNAs were described in *L. monocytogenes* to date.

## Classification and mechanistic concepts of asRNAs

Antisense RNA derives from promoters located on the complementary strand of a gene or operon they target. Reported asRNAs in *L. monocytogens* comprise a heterogeneous group of transcripts with significant variability in length (30 to thousands of nucleotides), differences in origin and mechanisms (Mandin et al., 2007; Toledo-Arana et al., 2009; Mraheil et al., 2011; Wurtzel et al., 2012).

**Figure 1:**
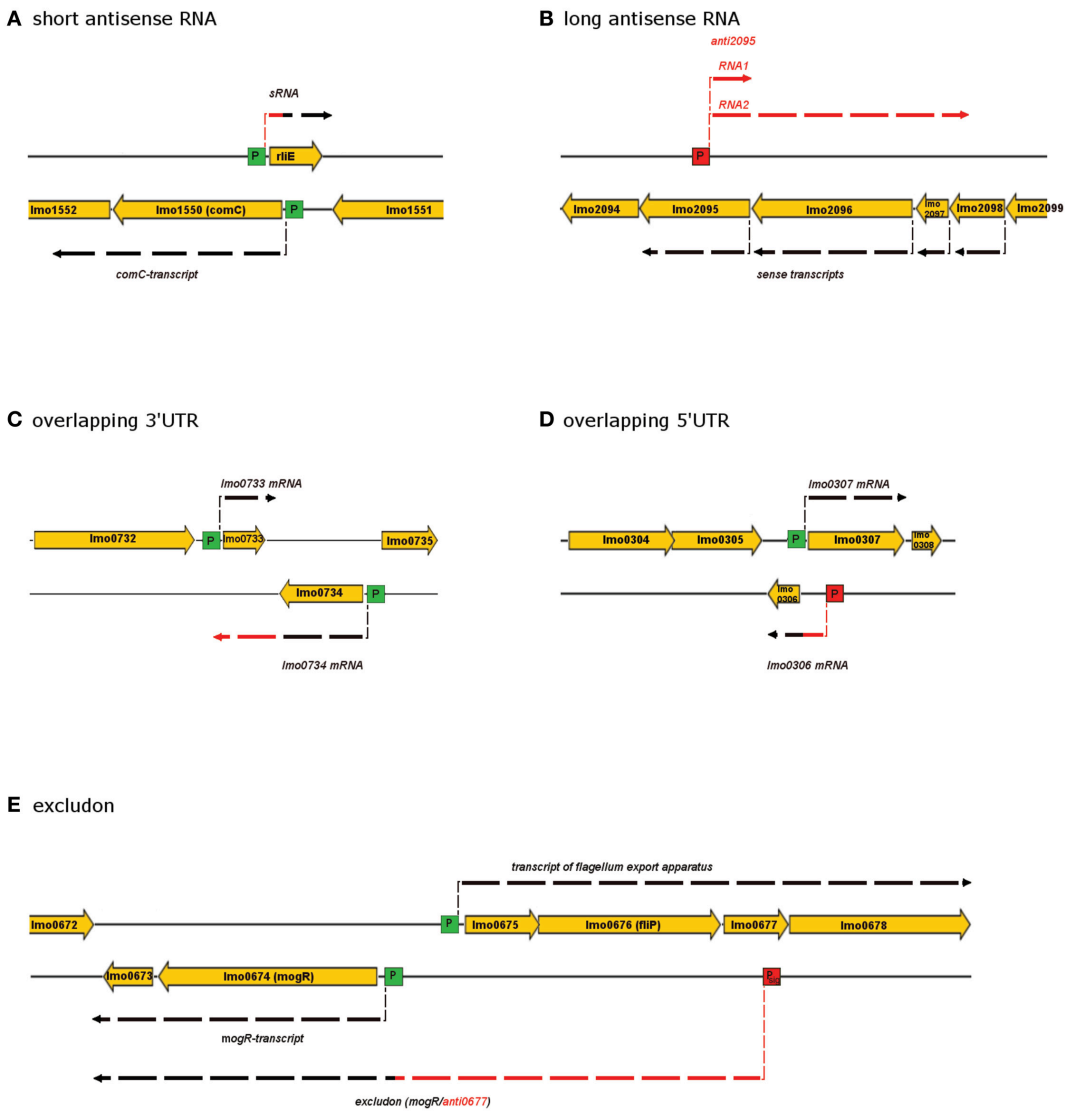
**Classes of *cis*-mediated antisense regulation found in *L. monocytogenes***. Genes are depicted as yellow arrows while transcripts are illustrated by dashed lines. Red color of dashed lines highlights regions antisense to other transcripts. Schematic views of a short antisense RNA regulation **(A)**, a long antisense RNA regulation **(B)**, overlapping 3′UTR **(C)**, overlapping 5′UTR **(D)**, and the excludon concept **(E)** are given.

According to these characteristics asRNAs can roughly be classified in five categories: (i) short, (ii) long, (iii) 3′UTR, (iv) 5′UTR, and (v) excludon (Figure 1).

Short asRNA that are antisense to genes in *L. monocytogenes* are for example rliE, rli23, rli25, rli29, rli30, and rli35 (Toledo-Arana et al., 2009).

Besides this, a remarkable example of sRNAs oriented antisense to each other was described for rli112. This sRNA is encoded in the intergenic region between *lmo2709* and *lmo2710* and is located antisense to the sRNA rli50 (Mraheil et al., 2011). Furthermore, another asRNA (rli28/29) is predicted to be antisense to rli78, which shares 94% homology with rli112 (Mraheil et al., 2011). To date, eight additional pairs or even groups of sRNA oriented antisense to each other have been described: rliC&rli125/rli85, rli42&sbrA/rli89, rli94&rli44, rliF&rli95, rli45&rli46, rli138&rli139, rli98&rli48, rli99&rli140 (Mandin et al., 2007; Toledo-Arana et al., 2009; Mraheil et al., 2011; Wurtzel et al., 2012).

Long asRNAs are transcripts of several hundred nucleotides that overlap more than one ORF. A representative of this class covers *lmo2095*–*lmo2098* (Toledo-Arana et al., 2009). Interestingly, using tiling array and northern blot analysis, two different antisense transcripts with the same transcription start side but alternative termination sites were detected. While one transcript (RNA1) was 255 nucleotides in length and located exclusively antisense to *lmo2095*, the second transcript (RNA2) was 2149 nucleotides in length and spans across neighboring genes partially including *lmo2098* (Figure 1B). The same study reported two other long asRNA that were slightly shorter but still span multiple ORFs (anti2095–2098 and anti2394–2395). Four additional potential long asRNAs (anti2046, anti2259, anti2677, und anti2717) overlapping to multiple ORFs were recently described (Behrens et al., 2014).

The concept of 5′-UTR overlapping asRNAs were found for some adjacent genes that are divergently oriented (transcription takes place in opposing direction starting from proximal promoters). It might represents an effective way to regulate neighboring genes. For example, transcription of *lmo0306* starts in the 5′UTR and thereby overlaps with the transcript of *lmo0307* (Figure 1D).

3′UTR asRNA are conceptually similar to 5′UTRs, however, the involved genes are located in a convergent orientation (distal promoters on opposite strands with converging transcription direction). For example, *lmo0733* and *lmo0743* both encoding putative transcription regulators, interact through 3′UTR (Figure 1C). The transcripts of *lmo0734* substantially overlap the ORF of the divergent oriented *lmo0733* with 750 nucleotides (Toledo-Arana et al., 2009). Thus, asRNAs deriving from both 5′- and from 3′UTR of adjacent genes exemplify a way to link the expression of two neighboring genes.

Most recently, a new antisense RNA-mediated concept of gene regulation was discovered in *L. monocytogenes*—the excludon (Wurtzel et al., 2012). An excludon is a remarkably long asRNA extending over multiple neighboring genes, which are organized in two sets—one set of genes being divergent orientated to the other (Figure 1E). The asRNA overlaps with one set of genes and thereby prevents expression of those by complementation, while it serves as a coding sequence for the other. Ipso facto, expression of the overlapping gene is inhibited, while expression of the opposite divergent gene is increased. Genes regulated by excludons often have related or opposite function, thus, it is most likely that excludons serve as asRNA-mediated biological switches (Wurtzel et al., 2012).

## Mechanisms of asRNA in *L. monocytogenes*

Although next generation sequencing was instrumental in the identification of several novel asRNAs in *L. monocytogenes*, precise mechanisms of action of asRNAs remain largely unknown. Based on limited mechanistic knowledge in *L. monocytogens* and mechanisms of asRNAs in other bacteria, some concepts have emerged. asRNA/target interactions can occur on different levels: (i) transcription, (ii) transcript stability, or (iii) translation.

On a transcriptional level, two mechanisms, transcription interference, and transcription attenuation were described. In transcription interference, the transcription of the target sequence is hindered by parallel transcription of the asRNA from a promoter locate opposite convergent from the sense promoter. The resulting asRNA is likely just a byproduct of this mechanism and the process of asRNA transcription itself rather than the intrinsic asRNA function represents the regulatory mechanism (Brantl and Wagner, 2000; Callen et al., 2004). In transcription attenuation sense transcription is prematurely stopped by a termination structure that forms upon interaction of the asRNA with the mRNA (Brantl and Wagner, 2000; Stork et al., 2007). To date, these mechanisms were confirmed in *Staphylococcus aureus, Streptococcus pyogenes*, and *Vibrio anguillarum* (Stork et al., 2007; Brantl and Bruckner, 2014), but not in *L. monocytogenes*.

asRNA-mediated alteration of transcript stability could occur by complementation with subsequent RNase-mediated degradation of the sense/antisense RNA duplex as shown in *Salmonella typhimurium, S. aureus*, and in *Synechocystis* sp. (Duhring et al., 2006; Lee and Groisman, 2010; Lasa et al., 2012). Although most asRNA/mRNA interactions are thought to result in degradation of the target sequence, asRNAs have also the potential to stabilize a sense transcript. Mechanisms involve the stabilization of transcripts by inducing cleavage of unstable polycistronic transcripts. A striking example of this case was demonstrated in *Escherichia coli* for *gadXW* (Opdyke et al., 2004, 2011; Tramonti et al., 2008).

Another stabilizing mechanism shown in *Prochlorococcus* sp. MED4 and *Synechocystis* sp. PCC 6803 functions via the masking of the RNases cleavage sites and thereby prevent degradation of a target transcript by formation of the asRNA/mRNA duplex (Stazic et al., 2011; Sakurai et al., 2012). So far, none of these regulatory mechanisms were demonstrated in *L. monocytogenes*.

Besides those mechanisms, some asRNA are supposed to also function in *trans*. Therefore, these transcripts can interact with genes encoded at different sites in the chromosome.

The asRNA rliE in *L. monocytogenes* is illustrative of this class. rliE overlaps with the gene *comC* and thereby likely acts as *cis*-regulator. In addition, as possible targets for rliE in *trans comEA-EB-EC, comFA-FC*, and *lmo0945* were found (Mandin et al., 2007). Similar to *comC*, all of these genes are putatively involved in competence, thus, rliE may represent a global regulator of this machinery.

At a more distal level, asRNAs can prevent translation by binding to the SD sequence of the target mRNA (Kawano et al., 2007). Inability of the ribosome to bind the SD region obstructs translation of the sense sequence.

## What are the main functions of antisense RNA in *Listeria monocytogenes*?

Reports on precise biologic functions of asRNAs in *L. monocytogenes* remain scarce and knowledge on asRNAs is mostly of descriptive nature. Reviewing functions of asRNA for bacteria it has been reported that antisense RNA regulation is frequently used for distinct purposes. In detail, asRNA is used to repress transcription of transposases or genes that encode for toxins as well as to control the expression of transcription regulators (Thomason and Storz, 2010). This is consistent with three asRNAs rli23, rli25, and rli35 described in *L. monocytogenes*, which overlap the transposase genes *lmo0172, lmo0330*, and *lmo0828*, respectively (Toledo-Arana et al., 2009). Furthermore, asRNAs that target transcription regulators are abundantly found in the *Listeria* genome, such as the above mentioned *lmo0733* and *lmo0734* (Figure 1C) (Toledo-Arana et al., 2009). In total, ~10% of all asRNA described for *L. monocytogenes* to date are thought to be involved in regulating transcription regulators.

Besides this, the well-investigated asRNA in *L. monocytogenes* are implicated in the control of metabolism, virulence, bacterial architecture and different transporting systems (Toledo-Arana et al., 2009; Mraheil et al., 2011; Wurtzel et al., 2012; Mellin et al., 2013; Behrens et al., 2014) and presage significant involvement of asRNAs in different domains of bacteria.

The best-established function was described for anti0677 controlling the flagellum biosynthesis excludon, which downregulates *lmo0675-0676-0677* encoding for the flagellum export apparatus and contributing to expression of the motility gene repressor MogR (*lmo0674*) (Toledo-Arana et al., 2009). The anti0677 promoter is responsive to the stress and temperature-activated transcription regulator RNA polymerase factor σ^B^. Temperature-induced MogR-mediated flagellum biosynthesis suppression was shown to be important for virulence of *L. monocytogenes* (Grundling et al., 2004). Although disputed in literature, flagellum expression has been suggested to induce the host inflammatory response (Hayashi et al., 2001). Thus, anti0677 inhibits expression of the flagellum export apparatus and promotes MogR expression and might thereby also contribute to abrogating the host response to *L. monocytogenes*.

Recently, Mellin et al. described a vitamin B12-binding riboswitch-regulated asRNA (Mellin et al., 2013). The *pocR* gene (*lmo1150*) encodes a transcriptional regulator, which activates transcription of the neighboring *pdu* and *cob* genes in the presence of propanediol. Pdu and Cob are essential for the catabolism of 1,2-propanediol catabolism and vitamin B_12_ biosynthesis. Propanediol is a byproduct of the metabolism of commensal intestinal bacteria. The ability to metabolize propanediol is important for pathogenicity and provides a survival advantage for bacterial during infection. In the process of propanediol catabolism vitamin B_12_ is required as a cofactor for involved enzymes. The reported asRNA anti1150 (*aspocR*) overlaps with the *pocR* gene. Interestingly, *aspocR* is controlled by a vitamin B_12_ dependent riboswitch that prematurely terminates transcription of *aspocR* in presence of vitamin B_12_ and thereby generates only a small transcript previously known as rli39. Subsequently, PCR-based experiments confirmed that *pocR* transcription was negatively regulated by *aspocR*. Additional experiments using ectopically transcribed *aspocR* showed inhibitory action in *trans* on *pocR* expression. These findings emphasize that the utilized mechanism is rather transcription attenuation or inhibition of translation than transcription interference or modulation of transcript stability in this case. Given that *pocR* is important for vitamin B_12_ biosynthesis, here antisense regulation seems to be rather a fine-tuning mechanism than an on- off-switch (Mellin et al., 2013).

Two further reported excludons, anti1846 and anti0605, affect the regulation of a permease-efflux pumps and a putative permease-efflux pump, respectively (Wurtzel et al., 2012). Notably, the promoter of the anti0605-controlled excludon is sigB responsive. These excludons might represent a biologic switch to change between cellular uptake and release of components based on the extracellular environment.

Another reported excludon (anti0424) is most likely involved in regulating central metabolic pathways in *L. monocytogenes*. As it spans two divergently oriented genes encoding for enzymes necessary for the usage of different carbon utilization, it might represent a possibility for a selective switching between those pathways (Wurtzel et al., 2012).

## Conclusion

Technological and methodological advances transformed the field of RNA-mediated gene regulation in bacteria and provided insight into an unexpected complexity. In *L. monocytogenes* hundreds of ncRNAs, including even more than hundred asRNAs possibly implicated in the regulation of 102 *Listeria* genes, were discovered to date.

This number seems rather low compared to the scope reported from other bacteria and will presumably rise with further studies. Yet, as recent findings in *L. monocytogenes* show the dependency of some antisense transcripts on transcription factors or even the absence of a metabolite, the importance of experimental conditions is highlighted.

Also despite the rather low extent of asRNAs reported to date, *L. monocytogenes* has proven to be a valuable model organism for studying asRNA regulation and given rise to novel discoveries like the excludon concept that could then be transferred to other bacteria.

It might be speculated that asRNAs in *L. monocytogenes* likely act through different mechanisms and could either function as an on-off switch or fine regulators of a particular network. Thereby, asRNAs might be involved in regulating metabolic processes, virulence and determinants of host inflammatory response. In addition, the impact of asRNA regulation is spread as many targets of antisense regulation then again affect the expression of other genes (e.g., transcriptional regulators).

However, our understanding about mechanisms and function remains limited to few individual transcripts. Mechanistic and functional validation of ncRNAs, including asRNAs, will shed further light into the extent of RNA-mediated regulation in bacteria. This understanding may then allow to develop new approaches for therapeutics.

### Conflict of interest statement

The authors declare that the research was conducted in the absence of any commercial or financial relationships that could be construed as a potential conflict of interest.
